# Movement consistency during repetitive tool use action

**DOI:** 10.1371/journal.pone.0173281

**Published:** 2017-03-09

**Authors:** Sandra Dorothee Starke, Chris Baber

**Affiliations:** School of Engineering, College of Engineering and Physical Sciences, University of Birmingham, Edgbaston, Birmingham, B15 2TT, United Kingdom; Duke University, UNITED STATES

## Abstract

The consistency and repeatability of movement patterns has been of long-standing interest in locomotor biomechanics, but less well explored in other domains. Tool use is one of such a domain; while the complex dynamics of the human-tool-environment system have been approached from various angles, to date it remains unknown how the rhythmicity of repetitive tool-using action emerges. To examine whether the spontaneously adopted movement frequency is a variable susceptible to individual execution approaches or emerges as constant behaviour, we recorded sawing motion across a range of 14 experimental conditions using various manipulations. This was compared to free and pantomimed arm movements. We found that a mean (SD) sawing frequency of 2.0 (0.4) Hz was employed across experimental conditions. Most experimental conditions did not significantly affect the sawing frequency, signifying the robustness of this spontaneously emerging movement. Free horizontal arm translation and miming of sawing was performed at half the movement frequency with more than double the excursion distance, showing that not all arm movements spontaneously emerge at the observed sawing parameters. Observed movement frequencies across all conditions could be closely predicted from movement time reference data for generic arm movements found in the Methods Time Measurement literature, highlighting a generic biomechanical relationship between the time taken for a given distance travelled underlying the observed behaviour. We conclude that our findings lend support to the hypothesis that repetitive movements during tool use are executed according to generic and predictable musculoskeletal mechanics and constraints, albeit in the context of the general task (sawing) and environmental constraints such as friction, rather than being subject to task-specific control or individual cognitive schemata.

## Introduction

Tool use is associated with highly developed abilities in various species [1], and the ways in which humans use tools has been referred to as ‘one of the most exciting issues in psychology’ [2]. Previous studies into tool use have investigated for example EMG activity for carpentry tasks [3], mechanical principles in the use of striking tools [4], functional mechanics of stone knapping [5,6], model solutions to the motor equivalence (or degrees of freedom) problem [7], the effect of inertial properties on perceived tool quality in hammering and poking [8], ergonomics and skill quantification in jewellery making [9] or the impact of tool design on the musculoskeletal system [10]. Research has also considered biomechanics of tool use in non-human species such as primates [11]. Tool use is also an interesting field from the perspective of system dynamics due to the interactions that may arise between tool, environment and user system; it is little explored how important each of these three components are in determining the final behaviour of the human-tool-environment system [1].

In the growing literature on human tool use, there are two broad perspectives to understand tool-using action: one perspective focuses on top-down, schema-driven control of action guided by stored neural representations of a tool’s properties and action sequences [12–14], which could be considered in terms of motor programs [15], respectively motor schemata [16]. Such a top-down perspective suggests that there exists a pre-defined specification of the movement pattern which is executed in its entirety after being triggered. If tool use was purely schema driven, this may suggest notable variation in the adopted movement patterns between different tool users, as each may conceptualise and approach the task differently. Within-participant variation ought to be small as long as contextual demands were kept constant if the same pre-defined program was executed repetitively. The bottom-up perspective, on the other hand, suggests that actions arise from the properties of the human-tool-environment system without strong need for centralized control. In broad terms, such approaches assume that a user can respond to the task by deriving the necessary motor control parameters on-the-fly based on the tool properties [2,8]. From such a perspective, ‘intelligence’ emerges from the interaction between components of a system [17]. Self-optimisation [18] and optimal feedback control [19] are popular concepts to explain the adoption of a specific and repeatable movement pattern: both rely on an iterative convergence on an ‘optimal’ state without an a priori movement schema. This optimal state could be minimal energy expenditure, with input to the feedback system continuously being updated through the bodily sensory system through physiological processes. The interactions between system components in the human-tool-environment system have also been considered in terms of the task’s affordance through ‘affordance matching’ [2,20]: affordances describe the ways in which items lend themselves to being used. Given that there is evidence for both top-down and bottom-up control in the experimental literature, these two concepts remain competitive explanations for the observed behaviour in the domain of tool use, with an emerging trend towards more evidence for bottom-up control. While the psychological and neuro-control literature are building up to a substantial body of work, to date biomechanical research into the fundamentals of spontaneously arising tool use behaviour in context of musculoskeletal dynamics is sparse. In this paper, we investigate–from a biomechanical perspective–whether tool use movement can be considered to emerge as a predictable, bottom-up consequence of the human-tool-environment system. Our assumption is motivated by findings for other cyclical activities with specific ‘signature’ frequencies such as walking or running. We hypothesized that spontaneous repetitive actions in tool use would result in a narrow range of movement cycle frequencies similar to these cyclical locomotor activities.

Research into repetitive actions has long scientific history. Work in the early 20^th^ century already reflected on the rhythmically consistent nature of the operation of tools [21,22]. Repetitive actions and movements are common when using for example hammers, saws, screwdrivers, planes, axes or adzes. However, there was limited attempt to provide an explanation for these observations. Research in biomechanics has shown that repetitive actions are generally performed at rates which minimize energy consumption [23–25]. It is for example widely known that the self-elected, preferred stride frequency during walking and running coincides with minimal metabolic energy expenditure [18,25]; the same holds true for hopping or bouncing [26,27]. In contrast, swinging the limbs at more than 2–3 Hz is energetically very inefficient for humans [18,24,28]. The narrow range of frequencies chosen spontaneously for many repetitive activities suggests physiological and anatomical constraints as the determinant of preferred movement cycle rates. This emphasizes the importance of passive dynamic mechanisms in understanding adopted movement frequencies [29]. For example, a movement rate which matches the resonant frequency of a ‘system’ is often spontaneously adopted for locomotor tasks [30] and results in near-minimal, or minimal, energy consumption [18,29]. High efficiency at resonance is linked to the interplay between the muscle tendon unit’s (MTU) active components (muscle fibre shortening) and passive components (tendons, ligaments and connective tissues) [31,32]. At resonance, a large proportion of length changes results from the passive, elastic properties of the MTU and muscle contractions are almost isometric and hence highly efficient [31]. Consequently, in repetitive actions such as bouncing on the toes, at resonance hardly any active work is required from the muscles [32]. In line with these findings, a study that investigated the effect of strength training on freely adopted walking, running and cycling frequencies found no change in frequency after training [33], lending further support to the importance of the passive dynamics of the segments involved in a movement task as well as muscle physiology which determines optimum contraction rates. Hence, movement cycle rates seem to converge on a frequency determined by the musculoskeletal system with the outcome being a stable, repeatable and energy-efficient movement pattern.

The primary aim of this study was to investigate spontaneously adopted movement cycle frequency during a simple, repetitive tool use in various experimental conditions. We chose the task of sawing as an example of repetitive tool using actions, and manipulated sawing conditions to determine whether the spontaneously adopted sawing frequency is situation-specific (suggesting individual schemata) or generic (suggesting generic systems mechanics).

## Materials and methods

### General rationale

**Fig 1:**
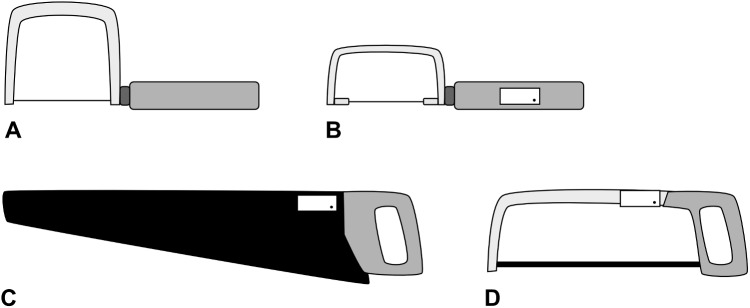
Schematic illustration of the four saws, drawn to scale. A–wood saw (experiment #1); B–piercing saw (experiment #2 and #3); C–hardpoint hand saw (experiment #3); D–hacksaw (experiment #4). The white rectangles depict a schematic illustration of the attached accelerometer unit for experiment #2 to #4.

This manuscript describes a series of five experiments, systematically manipulating multiple sets of variables which might have an influence on the adopted sawing frequency. Experiments were performed iteratively, where variables were chosen following the outcomes of the preceding experiment (for details on each experiment, please see individual paragraphs below). Four different saws were used across studies (Fig 1), with the ‘reference’ condition in each study being ‘normal’ horizontal sawing. Further, we added repeat conditions across and within studies to examine the robustness of our findings, such as repeating experiment #1 in experiment #4, and repeating the ‘normal’ condition at the end of a trial. Across experiments we changed the material which participants sawed into in order to vary the ‘environment’ factor: we assumed that the softer the material, the less damping would be added to the interacting oscillating system, and hence differences could be expected if the effect of material was large enough. Multiple techniques were used for data capture: after recording data for the first study using optical motion capture, we switched to accelerometers for less labour intensive data processing. For experiment #5, we used optical motion capture again (in a different facility to experiment #1) to quantify excursion distance.

### Recruitment

**Fig 2:**
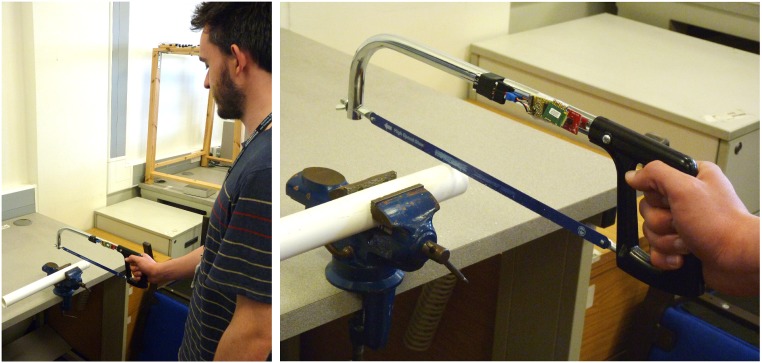
Data collection for experiment #4. Left: participant sawing into a plastic pipe, right: saw instrumented with accelerometer. The individual in this manuscript has given written informed consent (as outlined in PLOS consent form) to publish these case details.

For all experiments described in this paper, participation was voluntary and written informed consent was given by participants. Experiments were approved by the University of Birmingham Ethics Committee. Across experiments, participants were both male and female, ranging in age from 18 to 50 with most participants in their late teens and early twenties. The individual shown in Fig 2 in this manuscript has given written informed consent (as outlined in PLOS consent form) to publish these case details. Participants came from various backgrounds as detailed below. In experiment #3, a subset of participants comprised Engineering students taking part in the study for course credit. All participants were familiar with sawing, but had varying degrees of experience; none of the participants was considered an expert; rather, the results reported here are thought to represent a cross section of the normal population. The exception was experiment #2, where jewellery students from year 1 and 3 were recruited.

### Experiment #1: Horizontal and vertical sawing into wood

This experiment, comparing horizontal and vertical sawing, formed the pilot work for the present paper; hence the sample size was small. The experimental conditions are replicated in experiment #4 with a different saw / material and larger sample size; we present this pilot here to highlight the consistency of findings across experiments. In this experiment, we expected that vertical sawing would feel uncomfortable/unfamiliar and that participants would saw slower for this reason.

Five students from the University of Birmingham were asked to saw into a piece of wood by holding a wood saw (blade length: 13.4 cm, 15 teeth per inch, tooth height: approx. 1 mm) in two orientations: horizontally (as a familiar version of the task) and vertically (as an unfamiliar version of the task). All participants were right-handed. Horizontal and vertical sawing was performed in random order. The recording lasted as long as it took a participant to saw off a piece of the wood. An optical motion capture system (Oqus, Qualisys, Sweden) was used to track the position of a retro-reflective marker attached to the saw tip in 3D space at 200 Hz. The fixed global reference frame was defined as an orthogonal coordinate system aligned with the direction of sawing, with the positive z-axis pointing up along the line of gravity. The 3D coordinates of the marker were exported from Qualisys Track Manager (QTM, Qualisys, Sweden) for further processing in Matlab (The MathWorks, USA). In Matlab, the data range corresponding to continuous sawing effort was selected and displacement components for a single axis extracted: displacement along the y-axis (horizontal component) for horizontal sawing and displacement along the z-axis (vertical component) for vertical sawing.

### Experiment #2: Sawing discs and lines into copper

This experiment compared sawing a straight line to a circular shape and was performed as part of a study into dexterity in tool use [9]. In this experiment we tested whether a stable sawing frequency generalises to a different saw, task and material and a different, larger cohort of participants. We expected a difference between the two tasks only if the (conscious) control needed to rotate the material with one hand and the need to produce a specific shape would interact with the intuitively chosen sawing frequency.

Fifteen students from the first and third year of Birmingham City University’s School of Jewellery were asked to saw into a thin (approx. 1 mm) piece of copper. The task was to first saw straight lines (each approximately 1 cm in length) and second a circular shape (approximately 1 cm in diameter). The recording lasted as long as it took a participant to complete the task. In this experiment, a piercing saw (blade length 8.0 cm, 81 teeth per inch, tooth height 0.32 mm) was provided. All except one participant were right handed. Skill-levels varied due to prior experience, training and individual talent both within and across the two years. All participants sawed lines first, followed by a single circular shape. An instrumented saw, custom-built in our lab for the quantification of tool use [9], was used to record acceleration at 120 Hz. The handle contains a triaxial accelerometer mounted on the inside; the handle itself is formed by three equally spaced bars fitted with strain gauges which can be used to measure grip force. The local reference frame was defined as an orthogonal system aligned with the saw’s long-axis. Data from the accelerometer was recorded via Bluetooth through custom written software run on a nearby laptop and saved as.csv files containing raw accelerometer data (in mV). Data were imported into Matlab, where the acceleration component for the axis aligned with the saw’s handle was extracted. The data range corresponding to continuous sawing effort was selected for further processing, which included brief periods of rest in sawing motion during the disc sawing task while the disc was turned by hand.

### Experiment #3: Sawing with a large and small saw into wood

In this experiment we compared sawing with a large, coarse-toothed saw to the small saw used in experiment #2, again with a new participant cohort. We expected participants to use most of the available blade length for either saw, and to hence adopt a slower sawing frequency for the large saw compared to the small saw.

Two groups of participants were recruited from staff and students of the University of Birmingham. The first group consisted of 20 participants who were asked to saw into a piece of wood using the piercing saw from experiment #2 (blade length 8.0 cm, 81 teeth per inch, tooth height 0.32 mm). The second group consisted of 16 participants who were asked to saw into a piece of wood using a hardpoint hand saw (blade length 50 cm, 7 teeth per inch, tooth height approx. 2.5 mm). Participants were timed for approximately 15 to 20 seconds, as the hardpoint hand saw allowed sawing through the material quicker than the piercing saw and the set time aimed to match the two conditions. The instrumented piercing saw described in experiment #2 was used to record saw acceleration at 120 Hz. The hardpoint hand saw was instrumented with a tri-axial accelerometer unit attached to the blade near the handle together with the related circuit board and battery, recording data at 50 Hz. The board was equipped with a Bluetooth transmitter, and data were recorded and pre-selected as described in experiment #2.

### Experiment #4: Sawing under various conditions into a plastic pipe

In this experiment (Fig 2) we designed a larger number of conditions to test whether any other manipulation previously not tested might affect sawing frequency: we expected a lower frequency when requested to use a longer blade length, that sawing with either hand should not influence sawing frequency since musculoskeletal properties are almost identical and that adding weight to the saw would influence vertical sawing possibly causing a lower sawing frequency as a higher mass has to be moved by the same muscles.

Fifteen students from the University of Birmingham were asked to saw into a plastic pipe (35 mm diameter, wall-thickness 2 mm) using a hacksaw (blade length 28.5 cm, 24 teeth per inch, tooth height approx. 0.56 mm). All but three participants were right handed. In this experiment, the following experimental conditions were presented to each participant in random order: 1) sawing with the preferred hand without instructions (always performed first); 2) sawing with the non-preferred hand without further instructions; 3) sawing vertically with the preferred hand; 4) using precisely half the blade length (15 cm, limited by attachments to the blade) while sawing with the preferred hand and 5) using precisely the full blade length (28.5 cm) while sawing with the preferred hand. In addition, a further hacksaw was fitted with a 460 mm wrecking bar (crow bar) in order to increase its weight/inertia; this saw weighted 1.40 kg, while the regular saw weighted 0.24 kg. With this weighted saw, two further conditions were tested using the preferred hand: 6) sawing normally and 7) sawing vertically. After these seven conditions were completed, condition 1 (sawing with the preferred hand without instructions) was repeated. The whole session took approximately 15 minutes. Saws were instrumented with a tri-axial accelerometer unit attached to the blade near the handle as described for the hard point hand saw in experiment #3, recording data at 50 Hz. Participants were instructed to saw off a piece from the plastic tube; in case this took longer than approximately 30 seconds, participants were allowed to stop to prevent fatigue. Data were recorded and pre-selected as described in experiment #2.

### Experiment #5: Generic arm movements and mimed sawing

In experiment #5, we finally aimed to determine whether the spontaneously emerging sawing frequency corresponded to a frequency chosen for any horizontal arm movement to find out whether it was related to the general tool use task or emerged as some universally chosen arm movement rate. Further, this experiment was designed to quantify excursion distance.

Eight members of staff from the University of Birmingham were recruited to perform the following three tasks: 1) moving the arm forward and backward in a horizontal translation however they felt comfortable; 2) miming sawing, holding the hacksaw from experiment #4 and 3) sawing normally into a plastic pipe, repeating condition 1) from experiment #4. Conditions 1) and 2) were timed for 60 seconds. In condition 3), participants were asked to saw off multiple sections from the pipe, which took around 30 to 45 seconds. A retro-reflective marker was attached to the hand close to the carpometacarpal joint. An optical motion capture system (Bonita, Vicon, USA) was used to track marker position in 3D space at 100 Hz. The fixed global reference frame was defined as an orthogonal coordinate system aligned with the direction of sawing, with the positive z-axis pointing up in line with gravity. The 3D coordinates of the marker were exported from Vicon Tracker (Vicon, USA) for further processing in Matlab (The MathWorks, USA). In Matlab, the data range corresponding to continuous movement was selected. For the calculation of frequency, the horizontal displacement component in the direction of sawing was selected. For the calculation of sawing excursion, all three components were used.

### Data analysis

**Fig 3:**
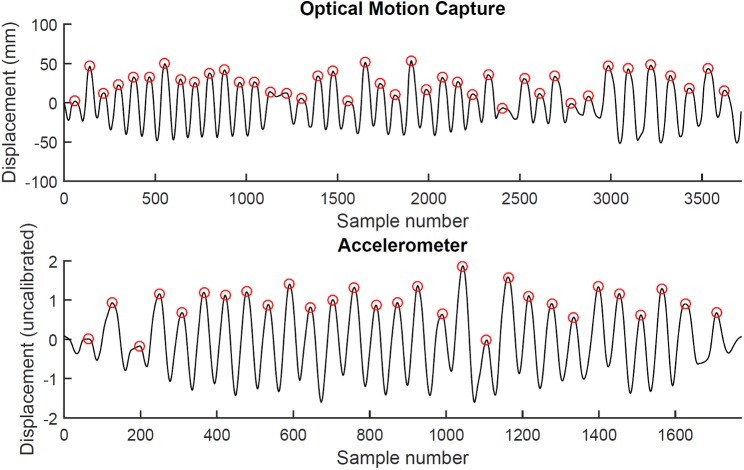
Signal processing. Maxima detection in the signals derived from optical motion capture (top) and an accelerometer (bottom). Each maximum (red circle) marks the start of a new sawing cycle, the time elapsed between maxima defines the sawing cycle duration.

Sawing cycle frequency was calculated based on the detection of maxima in displacement signals (Fig 3). The rationale for the approach was that sawing movement typically resembles a near-sinusoidal pattern, in which a sawing cycle can be defined as the duration from one specific event (such as a maximum) to the next. The signals extracted from optical motion capture and accelerometers were processed in a matching manner to ensure that results were comparable across experiments and data sources. Optical motion capture and accelerometer signals were offset-corrected for both methods by calculating the median value across the selected data range and subtracting it from the data. For the optical motion capture data (experiment #1), displacement along the primary axes of movement was highpass filtered with a 4^th^ order, zero-lag Butterworth filter (cut-off frequency 0.5 Hz) in order to match the filtering of the accelerometer data (see below) and then smoothed with a moving average filter (bin width: 10 frames) to avoid local extrema. Accelerometer data (experiment #2 to #4) were double-integrated and highpass filtered at each step with a 4^th^ order, zero-lag Butterworth filter (cut-off frequency 0.5 Hz) to eliminate drift arising from the integration procedure. This resulted in uncalibrated displacement trajectories. The resulting signal was smoothed with a moving average filter (bin width: 10 frames) in order to facilitate robust extrema detection as described above. For these signals, differences between adjacent data points were calculated and maxima detected as those frames where the sign of these differences changed from positive to negative in adjacent frames. In the rare cases where this did not work (usually due to local extrema), maxima were identified through manual input. The duration of each individual sawing cycle was calculated as the time between the identified maxima. Frequency was then calculated as the inverse of duration. For each participant, the median (to estimate central tendency) and interquartile range (IQR, to estimate within-participant variation) was calculated across all values, as distributions were sometimes skewed. Maxima timings were used to estimate the number of sawing cycles performed in each experiment by each participant.

Sawing excursion distance was calculated for each sawing cycle as the vector magnitude between the marker position at time t_1_ (coordinates [x_1_,y_1_,z_1_]) and time t_2_ (coordinates [x_2_,y_2_,z_2_]) based on maxima and minima identified from the single-axis trajectories as described above.

### Statistics

Statistical analysis was performed in IBM SPSS Statistics 22. Results were considered significant for P < 0.05 unless stated otherwise. Datasets for each experiment were tested for normality using the Shapiro-Wilk test. Since three datasets significantly differed from a normal distribution (studies 01V, 03S and 04NP, P ≤ 0.039), non-parametric statistics were performed. Statistics were performed for each of the five experiments separately. We performed within-subject comparisons for experiment #1, #2, #4 and #5 and an independent samples test for experiment #3, since participants differed between sawing with the small and large saw. Hence, for experiment #1 and #2, a Wilcoxon signed-rank test was run, for experiment #3, a Mann-Whitney U test was run and for experiment #4 and #5, a Friedman test was run. If the Friedman test indicated a significant effect of the condition on sawing frequency, pairwise Wilcoxon signed-rank tests, corrected for multiple testing using the Bonferroni correction, were executed post-hoc to establish between which exact conditions the difference laid. Participant-specific median and IQR values were compared using these tests. Finally, a Kruskall-Wallis test was performed to test whether the different blade lengths had an effect across experiments. Five datasets were used for this comparison: Experiment #2—line sawing (short blade), experiment #3 –both (short and very long blade), experiment #4 - ‘normal’ condition (long blade) and experiment #5 –‘real sawing’ (long blade).

### Predicted movements

To examine whether the cycle frequencies observed in this study can be predicted from the used blade length, we used established ‘time standards’ from the Methods Time Measurement (MTM) literature [34] to calculate the expected duration of generic arm movements over distances defined by different blade lengths. These standards give the expected duration of different arm motions over various ranges for specific aims of the movement, such as grasping, moving or reaching. For the type ‘move’, case B (move object to approximate or indefinite location, weight < 1.1 kg), which is most similar to the sawing task, we fitted a power function (R^2^ = 0.999) to the tabulated and published data [34] in order to predict values expected for blade lengths and travelled distances in the different experiments.

## Results

**Fig 4:**
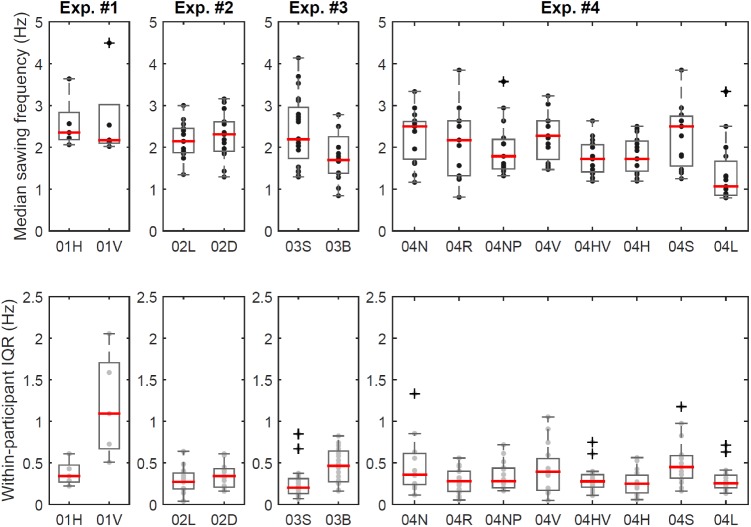
Results for experiment #1 to #4. Boxplots of participant-specific median in sawing frequency (top) and within-participant variation (bottom) for each experiment and each condition within the experiment. Black/grey dots: individual data points. Black/grey dots: individual data points. Experiment #1: 01H - horizontal sawing, 01V - vertical sawing; experiment #2: 02L - line sawing, 02D - disc sawing; experiment #3: 03S - small saw, 03B - large (big) saw; experiment #4: 04N - normal sawing, 04R –repeat of normal sawing at end of session, 04NP–sawing with non-preferred hand, 04V - vertical sawing, 04HV—vertical sawing with heavy saw, 04H - sawing with heavy saw, 04S - sawing with short blade length, 04L –sawing with long blade length.

In experiments #1 to #4 participants sawed with a consistent mean (SD) cycle frequency calculated across condition-specific median values of 2.0 (0.4) Hz. Median [IQR] sawing cycle frequency (Fig 4, top) was 2.35 [0.65] Hz for horizontal and 2.17 [0.92] Hz for vertical sawing in experiment #1, and 2.14 [0.58] Hz for line sawing and 2.31 [0.70] Hz for disc sawing in experiment #2. Conditions were not significantly different for either experiment #1 or #2 (P ≥ 0.733). Cycle frequency was 2.19 [1.22] Hz for sawing with the small saw and 1.70 [0.87] Hz for sawing with the large saw in experiment #3, being significantly different (P = 0.026). In experiment #4, sawing frequency ranged from 1.06 [0.81] Hz for sawing with a fixed-length long blade to 2.50 [1.19] Hz for sawing with a fixed-length short blade (see also Fig 4). Here the experimental condition had an effect on the sawing frequency (P < 0.001) as shown by a Friedman test. Pairwise post-hoc testing using Wilcoxon tests, however, was not able to detect a significant difference after correction for multiple testing between any of the conditions (P ≥ 0.011, adjusted alpha = 0.006). The different blade lengths had no significant effect on sawing frequency across the five experiments described in Material and Methods (P = 0.136).

Variation in sawing frequency (quantified as within-participant IQR) for a given participant and trial was low for most conditions (Fig 4, bottom): Median [IQR] within-participant variation was 0.43 [0.20] Hz for horizontal and 1.09 [1.03] Hz for vertical sawing in experiment #1, which was significantly different (P = 0.043). Variation was 0.27 [0.19] Hz for line sawing and 0.34 [0.22] Hz for disc sawing in experiment #2, not being significantly different (P = 0.155). Variation was 0.20 [0.18] Hz for sawing with the small saw and 0.46 [0.37] Hz for sawing with the large saw in experiment #3, being significantly different (P = 0.003). In experiment #4, variation ranged from 0.25 [0.15 / 0.21] Hz for sawing horizontally / with a fixed-length long blade to 0.45 [0.27] Hz for sawing with a fixed-length short blade. Here, the experimental condition had an effect on within-participant variation (P = 0.011) as shown by a Friedman test. Again however, pairwise post-hoc testing using Wilcoxon tests was not able to detect a significant difference after correction for multiple testing between any of the conditions (P ≥ 0.010, adjusted alpha = 0.006).

**Fig 5:**
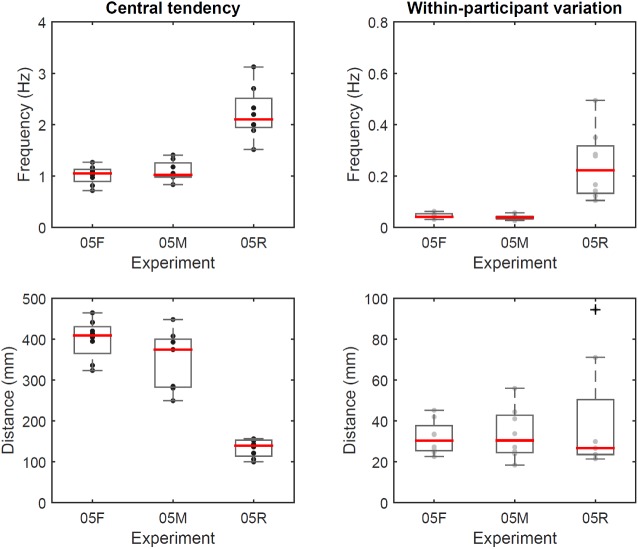
Results for experiment #5. Boxplots of sawing frequency (top) and excursion amplitude (bottom), showing participant-specific median values (left) and within-participant variation (right) in experiment #5. Black/grey dots: individual data points. 05F –free arm movements, 05M –mimed sawing, 05R –real sawing.

In experiment #5, median [IQR] cycle frequency was 1.05 [0.24] Hz for free arm movements, 1.02 [0.27] Hz for mimed sawing and 2.10 [0.57] Hz for the real sawing task (Fig 5, top). Median [IQR] excursion amplitude was 41.0 [6.5] cm for free arm movements, 37.5 [11.8] cm for mimed sawing and 14.0 [3.9] cm for the real sawing task (Fig 5, bottom). For sawing frequency, the condition had a significant effect (P = 0.001), where sawing frequency was significantly different between real sawing and free movement / mimed sawing (P = 0.012, adjusted alpha = 0.017). Matching this, the condition had a significant effect (P = 0.001) on the excursion amplitude, which was significantly different between real sawing and free movement / mimed sawing (P = 0.012, adjusted alpha = 0.017). Within-participant variation in movement frequency was 0.04 [0.01] Hz for free arm movement, 0.04 [0.01] Hz for mimed sawing and 0.22 [0.18] Hz for real sawing. Variation in excursion distance was 3.0 [1.2] cm for free arm movement, 3.0 [1.8] cm for mimed sawing and 2.7 [2.7] cm for real sawing. For within-participant variation in sawing frequency, the condition had a significant effect (P = 0.001), where sawing frequency was significantly different between real sawing and free movement / mimed sawing (P = 0.012, adjusted alpha = 0.017). Variation in excursion amplitude was not significantly different between conditions (P = 0.902).

When comparing the observed cycle frequencies to predictions made from generic arm movement times [34], we found a good match of 2.3 Hz predicted for a blade length of 8 cm (observed: 2.1 to 2.3 Hz) in experiment #2, 1.07 Hz predicted for 28.5 cm (observed: 1.06 Hz) in experiment #3 and 1.5 to 2.0 Hz predicted for 10 to 16 cm (observed: 1.7 Hz) in experiment #4. The only large discrepancy was noted for the constrained 15 cm blade length, with a prediction of 1.6 Hz and an observed frequency of 2.5 Hz. For the free and mimed arm movements in experiment #5, we again found a good match, predicting 0.9 Hz for the on average 40 cm excursion (observed: 1.0 Hz) during free arm movement / mimed sawing and 1.7 Hz for the on average 14 cm excursion (observed: 2.1 Hz) during real sawing.

## Discussion

Our results show that when sawing in unconstrained conditions, participants chose a sawing rhythm independent of most experimental manipulations. Used blade length appeared to be the only determinant of sawing frequency, albeit not always being significant at the pairwise level due to conservative testing. The hardpoint hand saw resulted in a small drop in operational frequency, likely in parts associated with the substantially higher and coarser distributed teeth of the saw. Observed movement matched expectations from generic arm movements [34], showing that the movement frequency and amplitude combination observed in sawing is in line with other, nonspecific arm movements executed over a similar distance. The only large exception observed for the 15 cm blade length (prediction: 1.6 Hz, observed: 2.5 Hz) was likely due to participants sawing in this condition by using the length limiting blocks as a point of collision instead of decelerating the saw through muscular effort, resulting in substantial noise. Our overall observed movement frequency of 2.0 Hz would correspond to a predicted average used blade length of 10 cm, which is in between the blade length of the piercing saw used in several tasks (8 cm) and the measured excursion distance for the hacksaw (14 cm); 10 cm is also in range of the suggested ‘used blade length’ evidenced by the collected sawdust in the teeth of the hardpoint hand saw (measured after experiment completion). These findings suggest that, while leaving room for small variation in the exact parameters, sawing is spontaneously executed at a robust combination of frequency and excursion distance. Material properties did not seem to cause systematic changes in sawing frequency: material varied from metal in experiment #2 to wood in experiment #3 and plastic in experiment #4 and #5, where for example the Young’s modulus (colloquial ‘elasticity’ or ‘stiffness’) of wood is a lot lower than metal (i.e. around 1 to 2 kN/mm^2^ radial or 11 to 14 kN/mm^2^ parallel to the fibre for wood compared to 125 kN/mm^2^ for copper [35]). How the observed convergence on a similar sawing pattern arises within the context of the nervous system and control of muscular contractions can only be speculated on. Broadly speaking, there are (at least) two routes for control of muscle activation: either a fast, learned / pre-programmed / feedforward pattern, or slower, feedback driven on-the-fly optimization using bodily sensing. Feedback control (as opposed to feed-forward control) has been proposed as the mechanism controlling oscillation frequency during bouncing [27], where the oscillation frequency is tuned to the resonance frequency of the oscillating system over several minutes. On the other hand, research investigating the contribution of pre-programmed and feedback-based processes to an adopted stride frequency and speed argues that both processes play a role, with a dominant contribution of the pre-programmed, fast process [36,37].

### Why 2 Hz?

Why do people spontaneously saw at 2 Hz? The answer may lie in intrinsic system dynamics of the musculoskeletal system tuned to e.g. preferred muscular contraction rates, but may additionally lie in the use of available cognitive resources. It has even been hypothesized that in fact the human system ‘resonates’ around a 2 Hz frequency [38]. We believe that performing repetitive tool use tasks at rates which match those found in locomotor tasks appears logical, as the musculoskeletal system can be assumed to already be optimized, or tuned, to perform regular locomotor tasks efficiently. Hence, cyclical muscle contractions should be efficient for such regularly performed rates. Humans commonly perform a variety of repetitive, or cyclical, movements such as walking, running, hopping and cycling at rates spanning approximately 0.8 to 3.0 Hz (see S1 Text and S1 Table, summarizing 16 studies reviewed for this paper). The interquartile ranges for sawing cycle frequency were bounded between around 1 and 3 Hz, matching this range. Further in line with these bounds, a model of cyclical muscle contractions suggests that when applied to type I and type IIA fibres, the predicted maximum power output occurs at around 1.5 Hz and 3.7 Hz, respectively [39] (although, as the authors point out, this is a slight over-estimate due to simplifying switch on/off times to zero). It has also been claimed that there is evidence for 2 Hz marking the threshold beyond which agonist and antagonist action cannot be fully separated for hand and wrist movements [40]. Cyclical action performed against substantial resistance, such as swimming and rowing, is typically performed at lower frequencies of approximately 0.3 to 1.3 Hz (see S1 Table, summarizing nine studies reviewed for this paper). Adding weight (0.24 kg vs. 1.40 kg) to the saw did not change the movement cycle frequency in our study, where additional weight may be compensated for by recruiting more muscle fibres. In Barnes (1980), the predicted duration for arm movements increases as a function of weight carried, however only for a mass > 1.13 kg.

The principles at the level of neural muscle activation may actually be similar to those in musculoskeletal dynamics: for example, a resonance model of the perceptual-motor system [38] peaked at a frequency of 1.8 to 2.0 Hz (0.50 to 0.55 s intervals), and neural entrainment centres around 2 Hz [41]. It is interesting to note that there exists differential control of slow and fast movements: previously, brain activation work has consistently observed a ‘rate effect’, where brain activation is dependent on the rate of movement in a differential manner (for summary see e.g. [42]). For movement frequencies ≥ 1.5 Hz in a finger tapping task, an automatic, program-like mode is active which is computationally ‘cheap’ (albeit increasing cost with increasing frequency), while for frequencies < 1 Hz, movement cycles are controlled individually through closed-loop feedback, with an increase in computational cost. This finding of possibly U-shaped brain activation curves as a function of movement frequency matches the U-shaped curves found for muscular energy expenditure during locomotor tasks at different speeds [25]. The rate effect in brain activation has been observed in both, fMRI studies [43] and EEG studies [42]. A change in control strategy at around 2 Hz has also been reported for the task of matching rhythms [44] or for movement frequencies in different manual tasks that do or do not require sensory-motor feedback [45] and has also been speculated about in early work on controlled arm movements [46]. Further, the control of eye movements when tracking sinusoidal signals changes at 2 Hz from target tracking to a static or erratic eye orientation [47,48]. These observations could suggest that repetitive movements are performed at a rate which not only minimizes muscular energy expenditure, but also minimizes brain activity in order to free up cognitive resources for parallel tasks. Performing repetitive movements at low cognitive cost would make sense, especially if matching preferred rates dictated by the bodily structures. In future, it would be interesting to examine whether this hypothesis holds and whether other repetitive tasks follow the same pattern.

### Difference between sawing and free arm movements

In experiment #5, arm movements without cutting into material were consistent across participants, despite being half the frequency of real sawing, and results for real sawing matched the findings from experiment #4 (condition 04N and 04R) for both central tendency and within-participant variation, showing that the study was repeatable. In context of the likely separation of control strategy towards 2 Hz described above, we assume that the low frequency for free arm movements was the result of non-automatic muscular control, while sawing was controlled automatically. Findings remained in line with what was expected for the frequency/displacement relationship of generic arm movements predicted from Methods Time Measurement data in Barnes (1980). The low variation in frequency both within and across participants for free arm movement and mimed sawing was striking and even more pronounced than during the sawing tasks. The reason for this convergence is not clear: it may be the result of optimal muscle recruitment, resulting in minimal energy expenditure and hence resulting in similar contraction rates across participants. Since movement frequency and amplitude differed between tasks that involved sawing into material and tasks without doing so, it seems unlikely that the adopted behaviour during sawing is the result of a generic movement pattern that may always be adopted when moving the arm horizontally. When moving the arm during free movement and miming, we noted that participants moved the arm forward towards almost complete extension, while joints remained flexed during sawing. Full extension of the arm provides a structural reference point, which would explain the low variation within and across participants, since participants had a similar height; for children for example, one would expect different behaviour due to the shorter segments and scaling effects. Further research would have to explore why the specific and repeatable behaviour was converged on during both, free arm movements and the real sawing tasks.

### Movement variability

The variability in sawing cycle frequency across participants was a little higher in this study compared to other studies exploring simple movement tasks. For example, standard deviations have been reported at 0.08 Hz for the preferred walking frequency [18], 0.1 Hz for the preferred hopping frequency [26] and 0.1 Hz for the preferred cycling frequency on a bicycle [49] have been reported. In our study, the average standard deviation across the 14 conditions was 0.7 Hz, similar to variation found in a more complex bouncing task [27,50] with a standard deviation of around 0.5 Hz. In contrast, the free arm moments in experiment #5 were executed with a low within-participant variation of 0.2 Hz compared to 0.6 Hz for sawing. The less frequent an activity is performed, the more it may cause variation across individuals, who may not use the optimal frequency straight away. In a study into walking, running and cycling, between-participant variation was observed to increase from walking to running to cycling [33]. It has also been speculated that patterns for walking and running are genetically ‘engrained’, whereas patterns for activities such as cycling are learned, resulting in less consistent behaviour [33]. In our study, the relatively short duration of the tasks, usually lasting less than a minute, may not have allowed enough time for all participants to converge on an ‘optimum’ movement pattern: in a study into bouncing, subjects spontaneously adopted the optimal frequency only after six minutes engagement in the task [27]. Variation in sawing frequency within participants was small with a median (IQR) across conditions of 0.4 (0.2) Hz. Some of this variation might have arisen from participants speeding up towards the last movement cycles when they were just about to saw off a piece of material. While speeding up for the last cycles might affect the within-participant variability estimate, it would not affect the calculated median and the related metrics and results, since from observation not more than around 25% of cycles would have been affected; in fact, after visual inspection, most trials are considered free of this bias.

### Outlook and generalisation

In the present study, we did not use domain experts; rather, we set out to investigate spontaneous behaviour based on a sample assumed to reflect experience levels in the general population, in order to arrive at results that generalise. This means that if experiments are replicated elsewhere with random participants, results should be reproducible. Expert tool users, such as carpenters, may learn to change the movement pattern from the pattern adopted spontaneously by novices to a pattern which may not minimise energy expenditure with respect to muscle recruitment but be more efficient with respect to the actual outcome of the activity, such as sawing through a piece of wood in the fastest time possible by for example using the whole available blade length. This would be interesting to explore in future. Handedness of participants is not considered a confounding factor, as participants were asked to use their preferred hand, and results from experiment #4 showed that sawing with the left or right hand did not have a significant effect on sawing frequency. How far do our findings extrapolate? As part of the recognition of David Attenborough’s achievements in light of his 90th birthday, BBC World News showed a collage of his famous television moments. As part of this clip collection, an orangutan is shown sawing with a hardpoint hand saw. We calculated the sawing frequency from this clip and it averages to a mean (SD) of 2.0 (0.2) Hz, matching our finding for humans.

## Conclusions

In a series of five experiments, we demonstrate that when acting freely, people will use a saw largely independent of the saw’s dimensions and situational circumstances at a preferred cycle frequency of around 2 Hz. Only in experiment #3 did a longer blade length result in a small but significant change in operational frequency, which may be due to the rough teeth of this particular saw. The longer blade length in experiment #4 and #5 did not result in an operational frequency significantly different from that of the short blades, with the used blade length ranging from 8 cm (limited by the saw) to 14 cm (observed for the 28.5 cm saw). Our findings match the selection of a preferred frequency and excursion amplitude found for example during locomotion [51]. In contrast to the explanations of tool-use that rely on stored representations or extraction of motor control parameters, we believe that our results lend support to the hypothesis that the passive dynamic behaviour of the musculoskeletal structures and optimal muscle recruitment determine the cycle rate. The observed movement might hence simply emerge as a general behaviour resulting from bodily constraints independent of the activity type. If this was the case, then the study of tools needs to be more aware of the interactions between bodily dynamics and control strategies, rather than assuming that tool use behaviour is the result of an individual’s cognitive approach to the task. Based on our findings of a consistent sawing frequency, we propose that—similar to other cyclical activities with specific ‘signature’ frequencies such as walking or running—repetitive actions in tool use result in a narrow range of movement cycle frequencies that do not require complex control strategies or motor learning, but emerge as a consequence of biomechanical constraints in context of the tool-environment-user system, which may act as a function of required external force based on environmental characteristics and the general task–such as sawing–to be executed.

## Supporting information

S1 Dataset Data used in this manuscript. (XLSX)

S1 Table Cycling frequencies for various activities. Tabulated selection of published frequencies referred to in S1 Text at which humans perform various terrestrial activities such as walking, running, hopping, bouncing or other activities such as cycling, swimming and rowing.
(DOCX)

S1 Text Literature summary of common preferred movement frequencies. This short review presents a summary of commonly observed movement frequencies in locomotor tasks.
(DOCX)

## References

[1] Baber C (2003) Cognition and tool use: Forms of engagement in human and animal use of tools: CRC Press.

[2] Osiurak F, Jarry C, Le Gall D (2010) Grasping the affordances, understanding the reasoning: toward a dialectical theory of human tool use. Psychological review 117: 517 10.1037/a0019004 20438236

[3] Hammarskjöld E, Harms-Ringdahl K, Ekholm J (1990) Shoulder-arm muscular activity and reproducibility in carpenters' work. Clinical Biomechanics 5: 81–87. 10.1016/0268-0033(90)90042-5 23916165

[4] Drillis R, Schneck D, Gage H (1963) The Theory of Striking Tools. Human Factors: The Journal of the Human Factors and Ergonomics Society 5: 467–478. 10.1177/001872086300500505 14101570

[5] Bril B, Rein R, Nonaka T, Wenban-Smith F, Dietrich G (2010) The role of expertise in tool use: skill differences in functional action adaptations to task constraints. Journal of Experimental Psychology: Human Perception and Performance 36: 825 10.1037/a0018171 20695702

[6] Rein R, Nonaka T, Bril B (2014) Movement Pattern Variability in Stone Knapping: Implications for the Development of Percussive Traditions. PLoS ONE 9: e113567 10.1371/journal.pone.0113567 25426630 PMC4245206

[7] Bullock D, Grossberg S, Guenther FH (1992) A self-organizing neural network model for redundant sensory-motor control, motor equivalence, and tool use. Proceedings of the International Joint Conference on Neural Networks (IJCNN): 91–96.

[8] Wagman JB, Carello C (2001) Affordances and Inertial Constraints on Tool Use. Ecological Psychology 13: 173–195.

[9] Baber C, Gunduz Cengiz T, Starke S, Parekh M (2015) Objective classification of performance in the use of a piercing saw in jewellery making. Applied Ergonomics 51: 211–221. 10.1016/j.apergo.2015.05.002 26154220

[10] Grant KA, Habes DJ (1993) Effectiveness of a handle flange for reducing manual effort during hand tool use. International Journal of Industrial Ergonomics 12: 199–207.

[11] Ishibashi H, Hihara S, Iriki A (2000) Acquisition and development of monkey tool-use: behavioral and kinematic analyses. Canadian journal of physiology and pharmacology 78: 958–966. 11100944

[12] Chao LL, Martin A (2000) Representation of manipulable man-made objects in the dorsal stream. Neuroimage 12: 478–484. 10.1006/nimg.2000.0635 10988041

[13] Vaesen K (2012) The cognitive bases of human tool use. Behavioral and Brain Sciences 35: 203–218. 10.1017/S0140525X11001452 22697258

[14] Johnson-Frey SH (2004) The neural bases of complex tool use in humans. Trends in Cognitive Sciences 8: 71–78. 10.1016/j.tics.2003.12.002 15588811

[15] Keele SW (1968) Movement control in skilled motor performance. Psychological Bulletin 70: 387–403.

[16] Schmidt RA (2003) Motor schema theory after 27 years: Reflections and implications for a new theory. Research quarterly for exercise and sport 74: 366–375. 10.1080/02701367.2003.10609106 14768837

[17] Davids K, Glazier P, Araújo D, Bartlett R (2003) Movement Systems as Dynamical Systems. Sports Medicine 33: 245–260. 12688825 10.2165/00007256-200333040-00001

[18] Holt KG, Jeng SF, Ratcliffe R, Hamill J (1995) Energetic Cost and Stability during Human Walking at the Preferred Stride Frequency. Journal of Motor Behavior 27: 164–178. 12736125 10.1080/00222895.1995.9941708

[19] Todorov E, Jordan MI (2002) Optimal feedback control as a theory of motor coordination. Nat Neurosci 5: 1226–1235. 10.1038/nn963 12404008

[20] Bach P, Nicholson T, Hudson M (2014) The affordance-matching hypothesis: how objects guide action understanding and prediction. Frontiers in Human Neuroscience 8: 254 10.3389/fnhum.2014.00254 24860468 PMC4026748

[21] Drillis RJ (1963) Folk Norms and Biomechanics. Human Factors: The Journal of the Human Factors and Ergonomics Society 5: 427–441. 10.1177/001872086300500502 14101567

[22] Bernstein N (1967) The Coordination and Regulation of Movements. London: Pergamon Press, Oxford.

[23] Ralston HJ (1958) Energy-speed relation and optimal speed during level walking. Internationale Zeitschrift für Angewandte Physiologie Einschliesslich Arbeitsphysiologie 17: 277–283. 10.1007/BF00698754 13610523

[24] Zarrugh M, Radcliffe C (1978) Predicting metabolic cost of level walking. European Journal of Applied Physiology and Occupational Physiology 38: 215–223. 648512 10.1007/BF00430080

[25] Hoyt DF, Taylor CR (1981) Gait and the energetics of locomotion in horses. Nature 292: 239–240.

[26] Jones GM, Watt DGD (1971) Observations on the control of stepping and hopping movements in man. The Journal of Physiology 219: 709–727. 5157598 10.1113/jphysiol.1971.sp009684 PMC1331655

[27] Merritt KJ, Raburn CE, Dean JC (2012) Adaptation of the preferred human bouncing pattern toward the metabolically optimal frequency. Journal of Neurophysiology 107: 2244–2249. 10.1152/jn.00984.2011 22298828

[28] Connick MJ, Li F-X (2014) Changes in timing of muscle contractions and running economy with altered stride pattern during running. Gait & Posture 39: 634–637. 23948332 10.1016/j.gaitpost.2013.07.112

[29] Holt KG, Hamill J, Andres RO (1990) The force-driven harmonic oscillator as a model for human locomotion. Human Movement Science 9: 55–68.

[30] Turvey MT, Schmidt RC, Rosenblum LD, Kugler PN (1988) On the time allometry of co-ordinated rhythmic movements. Journal of Theoretical Biology 130: 285–325. 3419184 10.1016/s0022-5193(88)80031-6

[31] Takeshita D, Shibayama A, Muraoka T, Muramatsu T, Nagano A, Fukunaga T et al (2006) Resonance in the human medial gastrocnemius muscle during cyclic ankle bending exercise. Journal of Applied Physiology 101: 111–118. 10.1152/japplphysiol.01084.2005 16497843

[32] Dean JC, Kuo AD (2011) Energetic costs of producing muscle work and force in a cyclical human bouncing task. Journal of Applied Physiology 110: 873–880. 10.1152/japplphysiol.00505.2010 21212245 PMC3075134

[33] Sardroodian M, Madeleine P, Voigt M, Hansen EA (2015) Freely chosen stride frequencies during walking and running are not correlated with freely chosen pedalling frequency and are insensitive to strength training. Gait & Posture 42: 60–64. 10.1016/j.gaitpost.2015.04.003 25943407

[34] Barnes RM (1980) Motion and Time Study Design and Management of Work: John Wiley & Sons, Incorporated.

[35] Mayr M (2003) Technische Mechanik. Muenchen: Karl Hanser Verlag.

[36] Pagliara R, Snaterse M, Donelan JM (2014) Fast and slow processes underlie the selection of both step frequency and walking speed. Journal of Experimental Biology 217: 2939–2946. 10.1242/jeb.105270 24902746

[37] Snaterse M, Ton R, Kuo AD, Donelan JM (2011) Distinct fast and slow processes contribute to the selection of preferred step frequency during human walking. Journal of Applied Physiology 110: 1682–1690. 10.1152/japplphysiol.00536.2010 21393467 PMC4182286

[38] van Noorden L, Moelants D (1999) Resonance in the Perception of Musical Pulse. Journal of New Music Research 28: 43–66.

[39] Medler S, Hulme K (2009) Frequency-dependent power output and skeletal muscle design. Comparative Biochemistry and Physiology Part A: Molecular & Integrative Physiology 152: 407–417. 10.1016/j.cbpa.2008.11.021 19101645

[40] Jagacinski RJ, Flach JM (2003) Control theory for humans: Quantitative approaches to modeling performance: CRC Press.

[41] Will U, Berg E (2007) Brain wave synchronization and entrainment to periodic acoustic stimuli. Neuroscience Letters 424: 55–60. 10.1016/j.neulet.2007.07.036 17709189

[42] Lutz K, Koeneke S, Wüstenberg T, Jäncke L (2004) Asymmetry of cortical activation during maximum and convenient tapping speed. Neuroscience Letters 373: 61–66. 10.1016/j.neulet.2004.09.058 15555778

[43] Jäncke L, Specht K, Mirzazade S, Loose R, Himmelbach M, Lutz K et al (1998) A parametric analysis of the `rate effect' in the sensorimotor cortex: a functional magnetic resonance imaging analysis in human subjects. Neuroscience Letters 252: 37–40. 9756353 10.1016/s0304-3940(98)00540-0

[44] Mayville MJ, Bressler LS, Fuchs A, Kelso SJA (1999) Spatiotemporal reorganization of electrical activity in the human brain associated with a timing transition. Experimental Brain Research 127: 371–381. 10480272 10.1007/s002210050805

[45] Kunesch E, Binkofski F, Freund H-J (1989) Invariant temporal characteristics of manipulative hand movements. Experimental Brain Research 78: 539–546. 2612597 10.1007/BF00230241

[46] Neilson PD (1972) Speed of response or bandwidth of voluntary system controlling elbow position in intact man. Medical and biological engineering 10: 450–459. 5074848 10.1007/BF02474193

[47] Leist A, Freund HJ, Cohen B (1987) Comparative characteristics of predictive eye-hand tracking. Human Neurobiology 6: 19–26. 3583842

[48] Pew RW, Duffendack JC, Fensch LK (1967) Sine-Wave Tracking Revisited. IEEE Transactions on Human Factors in Electronics HFE-8: 130–134.

[49] Brisswalter J, Hausswirth C, Smith D, Vercruyssen F, Vallier JM (2000) Energetically Optimal Cadence vs. Freely-Chosen Cadence During Cycling: Effect of Exercise Duration. Int J Sports Med 21: 60–64. 10.1055/s-2000-8857 10683101

[50] Raburn CE, Merritt KJ, Dean JC (2011) Preferred movement patterns during a simple bouncing task. Journal of Experimental Biology 214: 3768–3774. 10.1242/jeb.058743 22031741

[51] Bertram JEA, Ruina A (2001) Multiple Walking Speed–frequency Relations are Predicted by Constrained Optimization. Journal of Theoretical Biology 209: 445–453. 10.1006/jtbi.2001.2279 11319893

