# Supplementary material for: Movement consistency during repetitive tool use action
Source: PLoS One. 2017 Mar 9;12(3):e0173281. doi: 10.1371/journal.pone.0173281 (PMC5344383; doi:10.1371/journal.pone.0173281)
Supplement: S1 Table — Tabulated selection of published frequencies referred to in S1 Text at which humans perform various terrestrial activities such as walking, running, hopping, bouncing or other activities such as cycling, swimming and rowing. (DOCX) [file pone.0173281.s002.docx]

**S1 Table. Cycling frequencies for various activities.**

Selection of published frequencies referred to in S1 Text at which humans perform various terrestrial activities such as walking, running, hopping, bouncing or other activities such as cycling, swimming and rowing.

| **Activity** | **Frequency** | **Reference** |
| --- | --- | --- |
| Walking (0.8 and 2.2 m s^-1^) | 0.8 to 1.8 Hz | 1 |
| Walking (1.6 m s^-1^) | 0.9 Hz | 2 |
| Running (1.5 m s^-1^ to at 3.1 m s^-1^) | 1.3 Hz to 1.4 Hz | 3 |
| Running (2.5 m s^-1^) | 1.3 Hz | 4 |
| Running (up to 5.8 m s^-1^) | 1.7 Hz | 5 |
| Sprinting (100 m track) | 2.5 Hz | 6 |
| Sprinting (25 m track) | 2.2 Hz | 7 |
| Hopping (one leg) | 2.06 Hz | 8 |
| Hopping (one leg) | 2.03 Hz | 9 |
| Jumping (both legs) | 1.8 Hz | 10 |
| Jumping (both legs) | 1.9 Hz | 11 |
| Bouncing (small amplitude) | 3.0 Hz | 12 |
| Bouncing (small amplitude) | 2.6 Hz | 13 |
| Cycling (one full turn of the crank) | 1.1 to 1.7 Hz | 14 |
| Cycling (one full turn of the crank) | 1.3 Hz | 15 |
| Cycling (one full turn of the crank) | 1.5 Hz | 16 |
| Swimming (breast stroke) | 0.7 to 0.8 Hz | 18 |
| Swimming (breast stroke) | 1.2 to 1.8 Hz | 19 |
| Swimming (breast stroke) | 0.3 to 1.3 Hz | 23 |
| Swimming (front crawl) | 0.5 to 0.7 Hz | 20 |
| Swimming (front crawl) | 0.4 to 0.7 Hz | 21 |
| Swimming (front crawl) | 0.9 to 1.0 Hz | 22 |
| Swimming (front crawl) | 0.3 to 1.3 Hz | 23 |
| Swimming (butterfly) | 0.5 to 1.2 Hz | 23 |
| Swimming (back stroke) | 0.3 to 1.1 Hz | 23 |
| Rowing | 0.5 to 0.7 Hz | 24 |
| Rowing (2000 metre race) | 0.5 Hz | 25 |

**References**

1 Loring, S. H., Mead, J. & Waggener, T. B. Determinants of breathing frequency during walking. *Respiration Physiology* **82**, 177-188 (1990).

2 Holt, K. G., Jeng, S. F., Ratcliffe, R. & Hamill, J. Energetic Cost and Stability during Human Walking at the Preferred Stride Frequency. *Journal of Motor Behavior* **27**, 164-178 (1995).

3 Cavagna, G. A., Mantovani, M., Willems, P. A. & Musch, G. The resonant step frequency in human running. *Pflugers Archiv : European journal of physiology* **434**, 678-684 (1997).

4 Farley, C. T. & González, O. Leg stiffness and stride frequency in human running. *Journal of Biomechanics* **29**, 181-186 (1996).

5 Cavagna, G. A., Willems, P. A., Franzetti, P. & Detrembleur, C. The two power limits conditioning step frequency in human running. *The Journal of Physiology* **437**, 95-108 (1991).

6 Kunz, H. & Kaufmann, D. A. Biomechanical analysis of sprinting: decathletes versus champions. *British Journal of Sports Medicine* **15**, 177-181 (1981).

7 Hunter, J. P., Marshall, R. N. & McNair, P. J. Interaction of step length and step rate during sprint running. *Med Sci Sports Exerc* **36**, 261-271 (2004).

8 Jones, G. M. & Watt, D. G. D. Observations on the control of stepping and hopping movements in man. *The Journal of Physiology* **219**, 709-727 (1971).

9 Austin, G. P., Tiberio, D. & Gaerett, G. E. EFFECT OF FREQUENCY ON HUMAN UNIPEDAL HOPPING. *Perceptual and Motor Skills* **95**, 733-740 (2002).

10 Farley, C. T., Blickhan, R., McMahon, T. A. & Taylor, C. R. Mechanics of human hopping. *Journal of Biomechanics* **20**, 896 (1987).

11 Dyhre-Poulsen, P., Simonsen, E. B. & Voigt, M. Dynamic control of muscle stiffness and H reflex modulation during hopping and jumping in man. *The Journal of Physiology* **437**, 287-304 (1991).

12 Dean, J. C. & Kuo, A. D. Energetic costs of producing muscle work and force in a cyclical human bouncing task. *Journal of Applied Physiology* **110**, 873-880 (2011).

13 Merritt, K. J., Raburn, C. E. & Dean, J. C. Adaptation of the preferred human bouncing pattern toward the metabolically optimal frequency. *Journal of Neurophysiology* **107**, 2244-2249 (2012).

14 Marsh, A. & Martin, P. E. Effect of cycling experience, aerobic power, and power output on preferred and most economical cycling cadences. *Medicine & Science in Sports & Exercise* **29**, 1225-1232 (1997).

15 Brisswalter, J., Hausswirth, C., Smith, D., Vercruyssen, F. & Vallier, J. M. Energetically Optimal Cadence vs. Freely-Chosen Cadence During Cycling: Effect of Exercise Duration. *Int J Sports Med* **21**, 60-64 (2000).

16 Argentin, S. *et al.* Relation between preferred and optimal cadences during two hours of cycling in triathletes. *British Journal of Sports Medicine* **40**, 293-298 (2006).

17 Sardroodian, M., Madeleine, P., Voigt, M. & Hansen, E. A. Freely chosen stride frequencies during walking and running are not correlated with freely chosen pedalling frequency and are insensitive to strength training. *Gait & Posture* **42**, 60-64 (2015).

18 Garland Fritzdorf, S., Hibbs, A. & Kleshnev, V. Analysis of speed, stroke rate, and stroke distance for world-class breaststroke swimming. *Journal of Sports Sciences* **27**, 373-378 (2009).

19 Pansiot, J., Lo, B. & Yang, G. Z. in *Body Sensor Networks (BSN), 2010 International Conference on.* 153-158.

20 Yanai, T. Stroke frequency in front crawl: its mechanical link to the fluid forces required in non-propulsive directions. *Journal of Biomechanics* **36**, 53-62 (2003).

21 Keskinen, K. L. & Komi, P. V. Stroking Characteristics of Front Crawl Swimming During Exercise. *Journal of Applied Biomechanics* **9** (1993).

22 Toussaint, H. Differences in propelling efficiency between competitive and triathlon swimmers. *Medicine & Science in Sports & Exercise* **22**, 409-415 (1990).

23 Craig, A. B. & Pendergast, D. Relationships of stroke rate, distance per stroke, and velocity in competitive swimming. *Med Sci Sports Exerc* **11**, 278-283 (1979).

24 Kleshnev, V. Stroke rate vs distance in rowing during the Sydney Olympics. *Australian rowing*, 2 (2001).

25 Hofmijster, M. J., Landman, E. H. J., Smith, R. M. & Knoek Van Soest, A. J. Effect of stroke rate on the distribution of net mechanical power in rowing. *Journal of Sports Sciences* **25**, 403-411 (2007).
